# Early Insights into COVID-19 in Persons Living with HIV and Cardiovascular Manifestations

**DOI:** 10.33696/aids.2.010

**Published:** 2020

**Authors:** Jelani K. Grant, Louis Vincent, Bertrand Ebner, Barry E. Hurwitz, Maria L. Alcaide, Claudia Martinez

**Affiliations:** 1 Department of Medicine, University of Miami Miller School of Medicine/Jackson Memorial Hospital, Miami, Florida, USA; 2 Behavioral Medicine Research Center, University of Miami, Miami, Florida, USA; 3 Department of Psychology, University of Miami, Coral Gables, Florida, USA; 4 Division of Endocrinology, Diabetes and Metabolism, Miller School of Medicine, University of Miami, Miami, Florida, USA; 5 Department of Medicine, Division of Infectious Diseases, University of Miami Miller School of Medicine, Miami, Florida, USA; 6 Department of Medicine, Division of Cardiovascular Disease, University of Miami Miller School of Medicine, Miami, Florida, USA

## Abstract

Persons living with HIV-1 (PLHIV) are at increased risk of cardiovascular complications in part due to the persistent inflammatory state despite viral suppression. SARS-CoV-2, the virus causing COVID-19, was declared a pandemic virus in March 2020, and caused over 30 million cases and 900,000 deaths worldwide to date. Individuals with COVID-19 are manifesting acute cardiovascular complications because of the inflammatory response associated with SARS-CoV-2 infection. It is not yet known whether having COVID-19 in the context of ongoing HIV-1 infection results in worse cardiovascular complications than in PLHIV who have not had COVID-19 infection. In this review, the potential for exacerbated cardiovascular manifestations in persons coinfected with HIV-1 and COVID-19 is considered.

## Introduction

At the end of 2019, a novel coronavirus was identified as the cause of a cluster of pneumonia cases in Wuhan, a city in the Hubei Province of China [1]. The virus was designated severe acute respiratory syndrome coronavirus-2 (SARS-CoV-2), with coronavirus disease (COVID-19) as the disease caused by the virus [2]. At the beginning of September 2020, globally there are more than thirty million confirmed COVID-19 cases [3], with a fatality rate ranging from 1.4% in mainland China to 7.2% in Italy [3]. Advanced age, underlying co-morbidities and immunocompromised states serve as significant risk factors for severe infection with SARS-CoV-2 [4–8]. Patients with SARS-CoV-2 infection can manifest a variety of cardiovascular complications including myocardial dysfunction, acute coronary syndrome, thromboembolic complications including pulmonary embolism leading to right heart failure among others [9–11]. SARS-CoV-2 infection is caused by binding of the viral surface spike protein to the human angiotensin-converting enzyme 2 (ACE2) receptor following activation of the spike protein by transmembrane protease serine 2 (TMPRSS2) [12]. ACE2 is expressed in the lung (principally Type II alveolar cells) and appears to be the predominant portal of entry [13]. ACE2 is highly expressed in the heart as well, counteracting the effects of angiotensin II in states with excessive activation of the renin-angiotensin system such as hypertension (HTN), heart failure (HF) and atherosclerosis [14]. Therefore, patients with these conditions are thought to be at higher risk for severe COVID-19 manifestations. In addition, SARS-CoV-2 infection drives the progression of systemic inflammation and immune cell overactivation, which may lead to a ‘cytokine storm’ and elevated levels of inflammatory cytokines, such as interleukin (IL)-6, IL-7, IL-22, which have been associated with oxygen radical formation and cardiovascular injury [15]. Indeed, autopsy studies have noted detectable SARS-CoV-2 RNA (and, in some cases, antigen) in the heart, in addition to respiratory tract specimens [16,17].

At present, over 1.4 million persons in the US are living with the Human Immunodeficiency Virus-1 (HIV) infection [18]. The southern US is disproportionally affected by HIV, accounting for greater than 15 cases per 100,000 persons [19–21]. The advent of anti-retroviral therapy (ART) has led to an increased life expectancy in PLHIV and recent estimates indicate that over 50% of PLHIV in the US are older than 55 years of age [22]. Aging and the status of immune activation and inflammation associated with chronic HIV, results in an increase of non-HIV comorbidities such as HTN, diabetes mellitus (DM), coronary artery disease, hyperlipidemia, and metabolic syndrome [23–25]. In fact, epidemiological evidence indicates that cardiovascular disease (CVD) is the leading cause of death among PLHIV in the US [26–29]. Although evidence of the pathophysiological effects of COVID-19 has rapidly emerged, research pertaining to the natural history of COVID-19 pathophysiology in the context of HIV-1 spectrum disease is scant. The increased prevalence of CVD and immunocompromised state in PLHIV suggest that PLHIV may experience worsened disease progression if infected with SARS-CoV-2. In addition, studies suggesting beneficial effects of ART, and the use of antivirals such as Remdesivir underscore the importance of evaluating the potential interactions between COVID-19 and HIV-1 infections and the risk of deleterious cardiovascular outcomes.

### Cardiovascular Manifestations of COVID-19

The frequency of myocardial injury, as reflected by elevation in cardiac troponin levels, is variable among hospitalized patients with COVID-19, with reported frequencies of 7 to 28 percent [9,30,31]. Of 416 patients with COVID-19 who were hospitalized in Wuhan, China, 19.7% had high-sensitivity troponin I (hs-TnI), an index of myocardial injury, above the 99th percentile upper reference limit on admission. Cardiac injury, as indexed by hs-TnI levels, were associated with a higher mortality compared to those without cardiac injury (42 of 82 [51.2%] vs 15 of 334 [4.5%]; p<0.001) [30]. Patients with elevated hs-TnI were older and had more comorbidities (including chronic heart failure in 14.6 versus 1.5 percent) and complications (acute respiratory distress syndrome, acute kidney injury, electrolyte disturbances, hypoproteinemia and coagulation abnormalities) compared with those without myocardial injury. The mechanisms underlying myocardial injury in these cases are not yet understood; however, in keeping with other severe respiratory illnesses, direct (“non-coronary”) myocardial damage may also play a role [32]. Hypoxia and electrolyte abnormalities, both known to contribute to the development of acute arrhythmias, have been frequently reported in the acute phase of severe COVID-19 illness [32]. However, the exact contribution of COVID-19 infection to the development of arrhythmias remains unknown [33]. In a multinational cohort of 8,910 patients from Asia, Europe, and North America, 304 patients (3.4 percent) had a history of arrhythmia, this prevalence was more than twice as common in patients who died from COVID-19 (6.8 versus 3.2 percent among survivors) [33]. Studies thus far on COVID-19 hospitalizations, document the presence of cardiac arrhythmias in 17 percent of the these entire cohort of patients, but 44 percent of patients admitted to an intensive care unit displayed arrhythmias [34]. The most common arrhythmia in patients with COVID-19 was sinus tachycardia followed by atrial fibrillation, atrial flutter, and monomorphic and polymorphic ventricular tachycardia [35].

The incidence of CAD following COVID-19 infections has been between 4.2 and 25 percent in Chinese reports [8,34,36]. Type I myocardial infarctions, due to the potential inflammatory and cardiotoxic mechanisms mentioned above, have been postulated to occur from atherosclerotic plaque rupture [37]. However, it appears that with COVID-19 infection, the majority of myocardial infarctions are type 2 and related to the primary infection, hemodynamic, and respiratory derangement [38]. There have been reports of increased coronary artery thrombus burden in patients with ST elevation myocardial infarction (STEMI), which is consistent with an increased frequency during the pandemic of thrombotic strokes, particularly in young people [39]. Alterations in the coagulation system, abnormal platelet function, or abnormal vascular endothelial function have also been postulated as possible mechanisms [40]. Some COVID-19 case reports have described findings consistent with a diagnosis of “clinically suspected myocarditis” or possible stress cardiomyopathy [41–45]. However, COVID-19 is likely a potential new cause of viral myocarditis, with studies reporting interstitial mononuclear inflammatory infiltrates or cardiac cell necrosis not typical of ischemic injury [46,47]. However, pathological studies have demonstrated changes in multiple organs, not only in lungs and heart but also liver and kidney.

In the previous Severe Acute Respiratory Syndrome (SARS) outbreak, chloroquine (CQ) and hydroxychloroquine (HCQ) were reported to have anti-SARS-CoV activity *in vitro*, suggesting that it may be potentially beneficial the treatment of COVID-19 infections [48]. Previous studies suggested that CQ and HCQ may inhibit the coronavirus through a series of steps: (i) by changing the pH at the surface of the cell membrane and thus, inhibiting the fusion of the virus to the cell membrane, and (ii) by inhibiting nucleic acid replication, glycosylation of viral proteins, virus assembly, new virus particle transport, virus release and other processes to achieve its antiviral effects [49]. However, in the context of HIV, it has been postulated that the anti-HIV- effect of these medications results not from a direct effect on the infected cell but rather by the inhibition of extracellular Tat effects [50]. Tat proteins not only promote the HIV immune dysregulation but also participates in the HIV-associated cardiovascular complications; hence whether CQ and HCQ would have cardioprotective effects in the context of HIV would be a consideration [51]. However in June 2020, the US Food and Drug Administration (FDA) revoked its emergency use authorization for these agents in patients with severe COVID-19 because data from randomized clinical trials suggest they provided no additional clinical benefit compared with placebo [52]. It is of relevance that other studies have shown some cardiotoxicities of CQ and HCQ resulting in QTc prolongation, other arrhythmias [53–56] and cardiomyopathies through direct lysosomal dysfunction via inhibition of lysosomal enzymes which could also be potentiated in the context of HIV [57,58]. Therefore, as of now the cardiovascular risks of CQ and HCQ appear to outweigh their benefits. Yet, further research is still necessary to clarify the role of these medications in HIV-Tat protein-cardiovascular disease pathway and its potential therapeutic benefits in the context of HIV.

## COVID-19 Co-infections in PLHIV

At the present time, there is limited data on the risk and severity of COVID-19 infection among PLHIV. However, the European AIDS Clinical Society reported that COVID-19 infection rates were similar in PLHIV compared to HIV-seronegative persons based on early reports [59–61]. As the number of cases of COVID-19 rise worldwide, these reports are clearly preliminary. Many challenges exist in estimating the prevalence of COVID-19 in PLHIV in the absence of global universal HIV testing and the absence of COVID-19 testing in patients not requiring hospitalization. This issue may also be complicated by the probable elevation of self-isolation in PLHIV due to heightened health concerns that their immune-compromised condition would render them more susceptible to more severe COVID disease course [62].

PLHIV have impaired immune responses even when on effective ART, and decreased responses to viral infections andvaccinations (as previously demonstrated with hepatitis B and influenza vaccines) [63–67]. The impaired immune responses may lead to higher risk and worse outcomes with any viral infection [68,69]. A small case series of PLHIV coinfected with COVID-19 in Turkey suggested that an understanding of the extent of comorbidities in PLHIV was essential in projecting mortality risk [70]. As case reports and small studies continue to provide insight into COVID-19 coinfections in PLHIV, recent reports from the United States and Uganda have highlighted atypical presentations of weakness, fatigue, diarrhea in the absence of cough or shortness of breath [71,72]. In Spain, a study of 47 PLHIV infected with COVID-19, found no difference in severity of disease or death [73]. Another case series in Germany with 33 coinfected patients found similar findings, with no increase in morbidity or mortality [74]. However, these findings should be viewed with caution because there was no direct comparison with HIV negative patients.

A cohort of 18 coinfected PLHIV from the United Kingdom, had significantly increased morbidity and mortality (27.8%) compared to HIV-negative counterparts [75]. Of note, PLHIV with COVID-19 requiring hospitalization were more likely to have significantly lower median CD4 count than those hospitalized without COVID-19. It has been posited that lymphopenia in the absence of T-cell activation may blunt the severe immunologic response seen in COVID-19 [76]. Furthermore, the incidence and severity in Spain of COVID-19 in 77,590 PLHIV receiving ART found that 236 patients were infected with COVID-19. Of these, 151 patients were hospitalized, 15 were admitted to the intensive care unit, and 20 subsequently died. These investigators reported that the risk for COVID-19 and related hospitalization was lower in PLHIV who were receiving tenofovir disoproxil fumarate (TDF)/emtricitabine (FTC). The authors hypothesized that nucleotide reverse transcriptase inhibitors (NRTI), such as TDF, might be effective against SARS-CoV-2 infection by inhibiting RNA-dependent RNA polymerase (RNAdRNAp) [77]. Overall, the role of preserved relative to compromised cellular immunity on the severity of COVID-19 disease course in PLHIV is very preliminary and requires further extensive evaluation to assess the role of ART in this context.

## Conclusion

In the midst of COVID-19 pandemic, and the potential risk to affect populations at risk, it is imperative to understand the extent to which COVID-19 may affect the health of PLHIV. Because PLHIV are now living longer and suffering of multiple comorbidities, there may be an increased risk of cardiovascular events in the setting of COVID-19. HIV infection and COVID-19 may independently lead to cardiovascular complications, but whether the co-infection provides an additive or interactive impact on cardiovascular complications is not known but is urgently needed.
